# Sedation Strategy, Procedural Comfort, and Clinically Detected Radial Artery Spasm During Transradial Coronary Angiography: A Prospective Observational Study

**DOI:** 10.3390/jcm15176783

**Published:** 2026-09-01

**Authors:** Uğur Karagöz, Senem Girgin, Mehmet Ali Coşar, Fahrettin Tuğrul Çitekçi, Tolunay Demirbaş, Ecem Büger, Büşra Tozduman, Sadık Volkan Emren, Murat Aksun

**Affiliations:** 1 Department of Cardiology, Faculty of Medicine, Atatürk Research and Training Hospital, Kâtip Çelebi University, Izmir 35620, Türkiye; fahrettin.citekci@saglik.gov.tr (F.T.Ç.); tolunay.demirbas@saglik.gov.tr (T.D.); sadikvolkan.emren@ikc.edu.tr (S.V.E.); 2 Department of Anesthesiology and Reanimation, Faculty of Medicine, Atatürk Research and Training Hospital, Kâtip Çelebi University, Izmir 35620, Türkiye; senem.girgin@ikc.edu.tr (S.G.); mehmetali.cosar@saglik.gov.tr (M.A.C.); ecem.buger@saglik.gov.tr (E.B.); murat.aksun@ikc.edu.tr (M.A.); 3 Department of Public Health, Faculty of Medicine, Atatürk Research and Training Hospital, Kâtip Çelebi University, Izmir 35620, Türkiye; busra.tozduman@ikc.edu.tr

**Keywords:** transradial angiography, radial artery spasm, propofol, monitored anaesthesia care, moderate sedation, procedural comfort

## Abstract

**Background/Objectives:** Radial artery spasm (RAS) is the most common periprocedural complication of transradial coronary angiography (TRA) and is identified clinically, from patient-reported forearm pain and operator-perceived catheter resistance, rather than by imaging. This study compared two sedation strategies used in routine clinical practice, namely fentanyl-midazolam moderate sedation (MS) and a propofol-integrated monitored anaesthesia care (MAC) regimen, with respect to procedure-related pain and the incidence of clinically detected radial artery spasm (cRAS). **Methods:** This single-centre, prospective, non-randomised, observational study enrolled 394 consecutive patients undergoing elective TRA (MS, *n* = 231; MAC, *n* = 163). cRAS was defined as at least two of five established criteria: three patient-reported pain items and two operator-perceived resistance items. No angiographic, ultrasonographic or physiological assessment of radial vasomotor tone was performed, and operators were not blinded. Co-primary endpoints were cRAS and post-procedural pain scores; the secondary endpoint was a composite of periprocedural complications. Factors associated with cRAS were analysed using multivariable logistic regression, including sensitivity analyses with decreasing dependence on patient-reported pain. **Results:** cRAS occurred in 59 patients (15%), 20.3% with MS and 7.4% with MAC (*p* = 0.001). VAS pain scores were lower with MAC (*p* < 0.001); complications did not differ (13.4% vs. 7.3%; *p* = 0.083). Female sex (odds ratio [OR] 3.60; 95% confidence interval [CI] 1.91–6.78), procedural duration (OR 1.04 per minute; CI 1.02–1.06) and MS rather than MAC (OR 3.69; CI 1.80–7.58) were independently associated with cRAS (all *p* < 0.001). The association persisted when the outcome was restricted to operator-assessed criteria (21.6% vs. 9.2%; OR 2.95; CI 1.55–5.59). **Conclusions:** The propofol-integrated regimen was associated with less procedural pain and less clinically detected spasm. These hypothesis-generating findings describe clinically apparent spasm and cannot establish prevention of true vasospasm. Within that limit, they support testing this strategy in a randomised trial with objective, blinded endpoints, particularly in women.

## 1. Introduction

Traditionally regarded as the gold standard for percutaneous coronary interventions, the transfemoral approach has been largely superseded by the emergence of transradial coronary angiography (TRA). By facilitating early mobilisation, maximising patient comfort, and substantially reducing access-site bleeding complications alongside short-term mortality, TRA has secured a Class I recommendation in current guidelines as the vascular access route of choice [1,2].

While the transradial approach has proven clinically superior to traditional transfemoral access, radial artery spasm (RAS) persists as its most prevalent periprocedural complication, affecting approximately one-third of patients, and recognised in routine practice from patient-reported forearm pain and operator-perceived catheter resistance rather than from imaging. This acute vasoconstrictive response not only mechanically impedes catheter manipulation and escalates the risk of procedural failure, often compelling a mandatory crossover to transfemoral access, but it also predisposes the vessel to endothelial injury and subsequent radial artery occlusion (RAO) [3].

The prophylactic administration of intra-arterial nitrates and calcium channel blockers to suppress local radial vasoreactivity has become a universal standard; nevertheless, the clinical incidence of spasm remains significant. This has prompted the use of systemic sedation strategies designed to modulate the triggering sympathetic tone at a central level [4]. In routine cardiological practice, moderate sedation based on a combination of fentanyl and midazolam offers a safe profile that limits sympathetic discharge by balancing analgesia and anxiolysis, and has been associated with lower reported rates of spasm [5]. In contrast to traditional opioid-benzodiazepine regimens, propofol-integrated protocols administered under the framework of Monitored Anaesthesia Care (MAC) have, in experimental preparations, been reported to relax vascular smooth muscle [6]. Whether this translates into a peripheral vascular effect during transradial procedures in patients has not been established. Despite the evidence in the literature suggesting that the use of sedation generally reduces spasm [4,5], few data compare agent-specific regimens with respect to procedural comfort and the spasm that is clinically detected in routine practice.

### Aims

The primary objective of this study was to compare a propofol-integrated MAC regimen with standard fentanyl-midazolam moderate sedation with respect to procedure-related pain and the incidence of clinically detected radial artery spasm (cRAS) during TRA. The secondary objective was to compare the periprocedural safety profiles of the two strategies. Notably, the study was not designed to evaluate whether either regimen prevents true radial vasospasm, as no angiographic, ultrasonographic, or physiological assessments of radial vasomotor tone were performed. Instead, the outcome was determined using clinical criteria that inherently include patient-reported pain. 

## 2. Materials and Methods

### 2.1. Patients and Study Design

This single-centre, prospective, non-randomised, observational study consecutively enrolled patients undergoing elective transradial coronary angiography at the Cardiology Clinic of İzmir Kâtip Çelebi University Atatürk Training and Research Hospital between January and June 2026.

**Figure 1:**
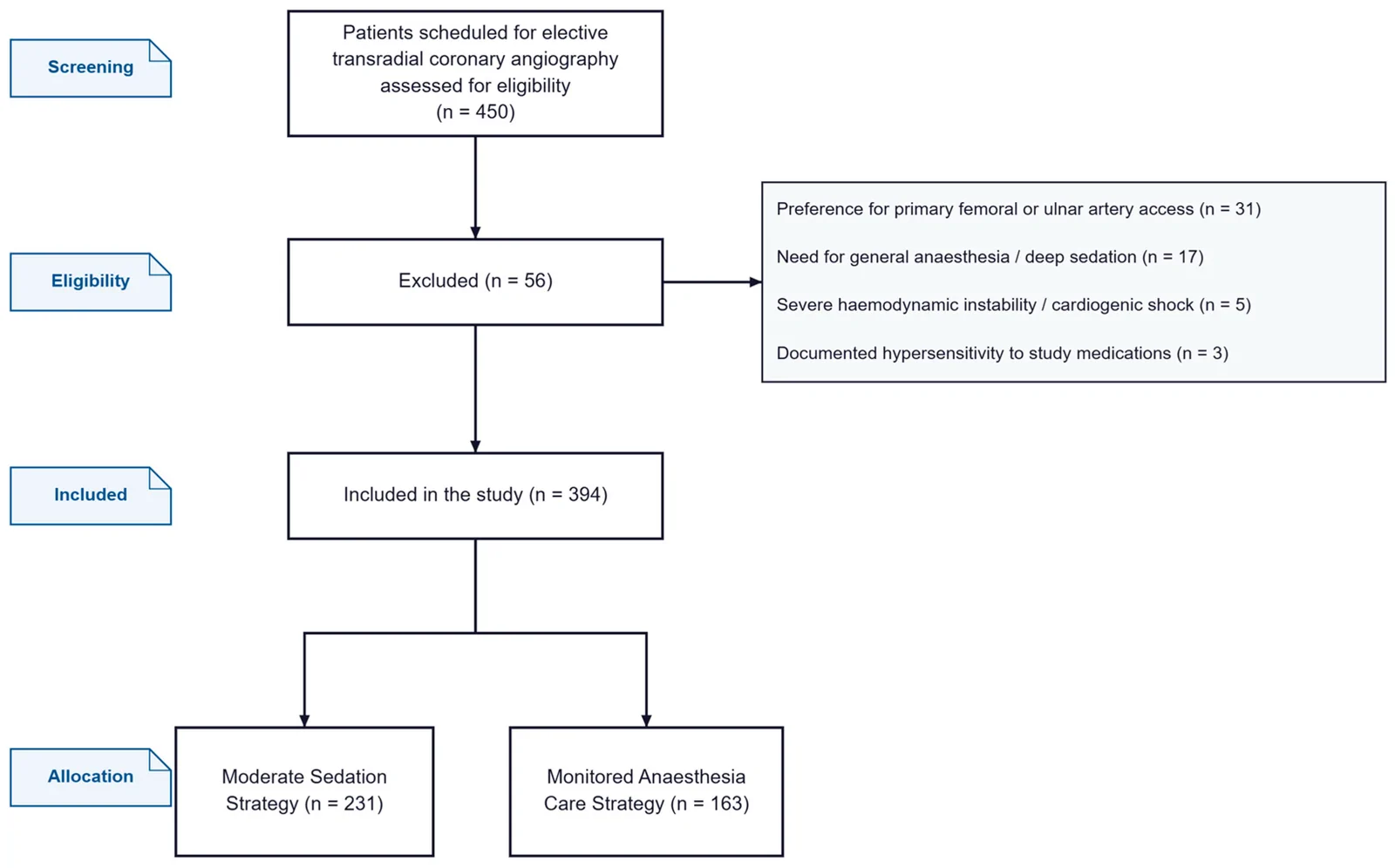
Study flowchart illustrating the patient screening, exclusion, and group allocation process.

The inclusion criteria were defined as follows: age 18 years or older, scheduled transradial coronary angiography (diagnostic and/or ad hoc percutaneous coronary intervention) utilising the radial artery as the arterial access route, and a level of cognitive cooperation sufficient to comprehend and respond to the 0–10 Visual Analogue Scale (VAS) for anxiety and pain assessment. Exclusion criteria included the need for general anaesthesia or deep sedation requiring airway instrumentation, preference for primary femoral or ulnar artery access, and the presence of severe haemodynamic instability (e.g., cardiogenic shock) requiring mechanical circulatory support or high-dose vasopressor therapy. Furthermore, patients presenting with haemodynamic collapse requiring active cardiopulmonary resuscitation, or those with documented hypersensitivity to the study medications, were excluded. The detailed patient screening, exclusion, and group allocation processes are illustrated in the study flowchart (Figure 1).

All eligible patients were enrolled consecutively to minimise potential selection bias and ensure a robust evaluation of the clinical outcomes of both routine sedation strategies. The assignment of patients to either sedation group was determined independently of the researchers, based on the evaluation of the attending anaesthesia team and the preference of the primary operator. Accordingly, patients were monitored under two main strategies:

1. Moderate Sedation (MS): Patients who received an intravenous (IV) bolus combination of 0.02–0.05 mg/kg midazolam and 0.5–1 µg/kg fentanyl prior to or during the procedure.
2. Monitored Anaesthesia Care (MAC): Patients who received a combination of propofol, midazolam, and fentanyl. In both study groups, pharmacological agents were titrated to achieve and maintain a Ramsay Sedation Scale score of 2 to 3 (patient cooperative, oriented, and tranquil). In this group, patients were administered a slow propofol loading dose of 0.5 mg/kg, followed by intermittent intravenous bolus increments of 10–20 mg based on the targeted sedation depth, or a maintenance infusion at a rate of 25–75 µg/kg/min according to clinical requirements. Regarding procedural timing, propofol induction in the MAC group was systematically administered prior to local anaesthesia infiltration and sheath insertion, mirroring the baseline sedation timing of the moderate sedation cohort.

Baseline demographic data (age, sex, body mass index) and medical history (hypertension, diabetes mellitus, hyperlipidaemia, peripheral artery disease, chronic kidney disease, smoking status) were recorded for all enrolled patients. Standard electrocardiography (ECG), pulse oximetry, and non-invasive blood pressure monitoring were maintained for all patients throughout the procedure.

In accordance with procedural standards, right or left radial artery puncture was performed utilising the traditional anatomical landmark (palpation) technique without routine ultrasound guidance. All procedures were performed by experienced interventional cardiologists (each having performed >200 prior transradial interventions). Following the local administration of 1 mL of 2% lidocaine at the access site, the number of puncture attempts was recorded. A 6-French radial sheath (Ares, Türkiye) was then inserted in all patients. Immediately following sheath placement, all patients received an intra-arterial administration of 300 µg of nitroglycerin and 2.5 mg of diltiazem as a standard vasodilator cocktail to prevent local radial artery spasm [7]. Under the anticoagulation protocol, a standard dose of 5000 IU of unfractionated heparin was administered to patients undergoing diagnostic coronary angiography alone. For patients undergoing percutaneous coronary intervention (PCI), the total heparin dose was adjusted to 70–100 IU/kg, based on the patient’s body weight. The procedural duration was defined as the time from local anaesthetic infiltration to radial sheath removal. The types and total number of catheters utilised were also recorded in the database. 

Prior to the procedure, written informed consent was obtained from all patients for the anonymised use of their clinical data within the scope of this research. The study protocol was approved by the Clinical Research Ethics Committee of İzmir Kâtip Çelebi University (Decision No: 2026-16), and all processes were conducted in strict compliance with the ethical principles outlined in the 1975 Declaration of Helsinki and its subsequent revisions.

### 2.2. Clinical Outcomes and Definitions

Patients’ pre-procedural anxiety levels were evaluated using the VAS for anxiety. Post-procedural pain was measured using the VAS for pain, recorded in the recovery area before transfer to the ward, once the patient was fully awake and oriented and the sedative effect had resolved. Accordingly, patients rated their experienced anxiety and feeling of discomfort on a scale from 0 (no anxiety/no pain) to 10 (unbearable anxiety/extreme pain). In the present study, the definition of cRAS was determined by the presence of at least two of the five core criteria established in accordance with standards currently accepted in the relevant literature [8]. These criteria are as follows:

1. Persistent forearm pain reported by the patient throughout the procedure;
2. Localised forearm pain occurring exclusively during catheter or guidewire manipulation;
3. Pain reported during the insertion or removal of the radial sheath;
4. The operator experiencing mechanical resistance during manipulation, felt as a strong grip on the catheter or guidewire by the artery (spasmodic entrapment), which prevents further advancement;
5. The operator detecting increased mechanical resistance against the removal of the radial sheath.

Cases meeting at least two of these five criteria during or after the procedure were classified as positive for cRAS. Three of the five criteria (1 to 3) are based on patient-reported pain, and two (4 and 5) rely on operator-perceived mechanical resistance. Because meeting two criteria was sufficient for case definition, a patient could in principle be classified as positive based solely on pain-related criteria. Throughout this report, the outcome is therefore termed clinically detected radial artery spasm (cRAS) to make explicit that it is a clinical construct derived from patient report and operator perception, rather than an imaging-verified or physiologically confirmed vasospasm. Neither angiography of the radial artery nor vascular ultrasound was performed, and no assessments of radial calibre, flow, or vasomotor tone were obtained at any point.

The real-time evaluation of the mechanical criteria and the elicitation of patient-reported symptoms were performed by the primary interventional cardiologist. Given the overt clinical manifestations of the distinct sedation protocols and the active presence of the anaesthesia team, the operating cardiologist could not be blinded to the group allocations. Consequently, both the patient-reported and operator-assessed components of this definition are susceptible to differential ascertainment bias between the groups, in a direction that would favour the MAC regimen.

The primary outcomes of the study are the development of cRAS during the procedure according to the specified criteria, and the post-procedural procedure-related pain score measured by VAS-Pain. The secondary outcomes were defined as the development of at least one periprocedural complication, such as hypotension (Mean Arterial Pressure < 65 mmHg), bradycardia (Heart rate < 50 bpm or clinically significant), respiratory depression (SpO2 < 90% or respiratory rate < 10 breaths per minute), or nausea/vomiting, with the objective of evaluating the systemic safety of the administered sedation and anaesthesia protocols.

### 2.3. Statistical Analysis

In the statistical analysis of the study data, the conformity of continuous variables to a normal distribution was evaluated using both visual methods (histograms and probability plots) and analytical methods (Kolmogorov–Smirnov or Shapiro–Wilk tests). Depending on their distribution characteristics, continuous variables were expressed as median and interquartile range (IQR; Q1–Q3: 25th–75th percentiles), while categorical variables were presented as frequencies (n) and percentages (%). In order to compare continuous variables between groups stratified by sedation strategies or the development of cRAS, the Mann–Whitney U test for independent groups was utilised. The comparison of categorical variables was performed using the Pearson Chi-square test or Fisher’s Exact Test, depending on the expected values in the contingency tables.

Multivariable logistic regression models were fitted to identify independent associations with each primary endpoint. For the first outcome, the multivariable logistic regression model incorporated variables demonstrating a statistically significant association with cRAS in the univariate analysis (*p* < 0.05) and clinically established baseline risk factors. For the second primary outcome, the post-procedural VAS-Pain score was initially dichotomised as ‘no pain’ (VAS = 0) versus ‘any procedure-related pain’ (VAS ≥ 1), given the highly skewed distribution of pain scores within our cohort, where the vast majority of patients reported no discomfort under effective sedation. Subsequently, a separate multivariable logistic regression model was constructed using the univariate variables that reached significance to identify the independent risk factors for procedural pain. Model calibration and goodness-of-fit for the regressions were assessed using the Hosmer–Lemeshow test.

Because both sedation regimens modify pain perception and reporting, the primary outcome definition is susceptible to differential detection between the study groups. Four analyses were performed to examine this, all after the primary analysis and reported as sensitivity analyses. First, the frequency of each of the five diagnostic criteria was compared between the sedation groups. Second, the proportion of patients meeting an operator-assessed criterion without fulfilling the composite definition was compared between groups; these represent the cases that the composite definition would predictably miss if deeper sedation suppressed pain reporting. Third and fourth, the multivariable model comprising sedation strategy, sex, and procedural duration was re-estimated for two endpoints independent of patient report: the presence of either operator-assessed criterion and the presence of both. These two endpoints do not represent alternative definitions of cRAS; rather, they serve as indicators independent of patient report to test whether the association between sedation strategy and the primary outcome is driven by its pain-based components. 

A deterministic bias analysis was performed to estimate the extent of differential detection required to generate the observed between-group difference assuming no true difference in vasoconstriction. Under the assumptions of identical true prevalence and perfect diagnostic specificity, the implied ratio of detection sensitivities was derived directly from the observed proportions.

The results of the analysis were reported as odds ratios (OR) with 95% confidence intervals (CI). All statistical analyses were performed with IBM SPSS Statistics version 27.0 (IBM Corp., Armonk, NY, USA), and a two-tailed *p*-value of less than 0.05 was considered statistically significant.

## 3. Results

A total of 394 patients undergoing transradial angiography were included in the study and divided into two groups according to the sedation protocol administered: moderate sedation (MS, *n* = 231) and monitored anaesthesia care (MAC, *n* = 163). With the exception of baseline oxygen saturation, no statistically significant differences were observed between the moderate sedation and MAC groups in demographic characteristics, comorbidity profiles or pre-procedural vasoactive medication (Table 1). Median saturation was 96.0% with MS and 97.0% with MAC (*p* = 0.001); both values lie within the normal range and a one-point difference at this level is not clinically meaningful, although it indicates that allocation was not wholly independent of baseline status.

Right radial access was utilised in the vast majority of cases (*n* = 379, 96.2%), with left radial access used in 15 patients (3.8%). The overall crossover rate to a transfemoral approach due to spasm was 2.5% (*n* = 10). Specifically, crossover occurred in 6 patients (2.6%) in the moderate sedation group and 4 patients (2.5%) in the MAC group, demonstrating no statistically significant difference between the strategies (*p* = 1.000). Procedural indications (diagnostic CAG or ad hoc PCI), number of punctures and catheters, and total procedure times showed no significant difference between the groups. Regarding pharmacological management, as anticipated, significantly lower doses of midazolam and fentanyl were used in the MAC group compared to the moderate sedation group (*p* < 0.001). In the MAC group, the median total propofol dose administered was 40.0 mg (IQR: 30.0–50.0 mg). Overall, 90 patients (55.2%) were successfully managed solely with a loading dose, whereas 73 patients (44.8%) required additional intermittent intravenous bolus increments of 10–20 mg or continuous maintenance infusion. The post-procedural VAS-Pain score, which was one of the primary outcome parameters of the study, was found to be statistically significantly lower in the MAC group (median 0.0) compared to the moderate sedation group (median 1.0) (*p* < 0.001) (Table 1). 

In the study population, cRAS was identified as the primary outcome, as defined by the established clinical criteria, in 59 patients (15%). Among these 59 cases, 56 patients (94.9%) exhibited at least one of the operator-assessed mechanical resistance criteria (severe catheter grip and/or increased resistance during sheath removal). An analysis of the demographic characteristics, vital signs, and baseline comorbidity profiles of the cases based on the development of cRAS revealed a high overall similarity between the groups (Table 2). Age, body mass index, and traditional cardiovascular risk factors were not significantly associated with cRAS. Women, in contrast, developed cRAS more often than men (33/136, 24.3% vs. 26/258, 10.1%; *p* < 0.001), and accounted for 55.9% of cRAS-positive compared with 30.7% of cRAS-negative patients.

Regarding the primary outcome, the choice of sedation strategy was significantly associated with cRAS (*p* = 0.001); the within-group incidence of cRAS was 20.3% (47 of 231) with moderate sedation and 7.4% (12 of 163) with MAC. The absolute difference was 13.0 percentage points (95% CI 6.4 to 19.5) and the unadjusted odds ratio for moderate sedation was 3.21 (95% CI 1.65 to 6.28) (Table 2). Furthermore, the preoperative anxiety levels of the patients (VAS-Anxiety) did not achieve statistical significance in relation to the development of cRAS (*p* = 0.057).

Regarding procedural parameters and clinical course, no difference was observed between diagnostic coronary angiography and ad hoc percutaneous coronary intervention with respect to the development of spasm. However, an increased number of catheter exchanges (*p* = 0.042) and a prolonged total procedural duration (*p* < 0.001) were significantly associated with the development of cRAS. Post-procedural VAS-Pain was higher in patients classified as cRAS-positive than in those classified as negative (median 3.0 versus 0.0; *p* < 0.001; Table 2). This comparison is not independent, since three of the five diagnostic criteria are themselves based on patient-reported pain, and it is reported for completeness rather than as a separate finding.

Secondary outcomes reflecting the periprocedural systemic safety of the applied sedation strategies (hypotension, bradycardia, respiratory depression, and nausea/vomiting) were detected in 43 patients across the entire population. When comparing safety profiles, no statistically significant difference was observed between the MAC strategy and standard moderate sedation regarding the development of composite secondary complications (7.3% vs. 13.4%, respectively; OR: 0.51, 95% CI: 0.26–1.03; *p* = 0.083; Table 3). Importantly, all observed respiratory and haemodynamic adverse events were mild and transient. Respiratory depression episodes were successfully managed with conservative measures, such as verbal/tactile stimulation or brief administration of supplemental oxygen. Similarly, hypotensive and bradycardic episodes were self-limiting or resolved rapidly with standard intravenous fluid boluses. None of the patients in either group required pharmacological reversal agents (e.g., flumazenil or naloxone), advanced airway instrumentation, or transfer to an intensive care unit.

Factors identified as being associated with cRAS in univariate analyses were entered into a multivariable logistic regression model to determine their independent effects on the development of spasm. The analysis identified female sex (OR: 3.60, 95% CI: 1.91–6.78; *p* < 0.001), prolonged procedural duration (OR: 1.04 for each 1 min increase, 95% CI: 1.02–1.06; *p* < 0.001), and the use of standard moderate sedation instead of monitored anaesthesia care (OR: 3.69, 95% CI: 1.80–7.58; *p* < 0.001) as independent factors associated with the development of cRAS (Table 4). Conversely, the number of catheter exchanges, which was found to be associated with spasm in univariate analyses, did not emerge as an independent risk factor in the multivariable model (for ≥3 exchanges vs. 0 exchanges, OR: 2.57, 95% CI: 0.61–10.78; overall *p* = 0.309). The multivariable logistic regression model demonstrated a good fit to the data, as indicated by the Hosmer–Lemeshow goodness-of-fit test (*p* = 0.86). Furthermore, the model explained 21.6% (Nagelkerke R^2^) of the variance in the development of cRAS.

The frequencies of all five individual diagnostic criteria were consistently lower in the MAC group (Table A1). Notably, pain-based criteria demonstrated greater relative reductions with MAC than mechanical resistance criteria. The two operator-assessed criteria also differed from one another: resistance at sheath removal was significantly less frequent with MAC, whereas spasmodic entrapment of the catheter or guidewire was not. The prespecified analysis of cases potentially missed by the composite definition further supported this observation. Of the patients with at least one operator-assessed criterion, 44 of 50 (88.0%) with MS and 12 of 15 (80.0%) with MAC also met the composite definition (Fisher’s exact *p* = 0.420). The remainder—patients with an operator-assessed finding who fell below the two-criterion threshold—accounted for 6 of 231 patients (2.6%) in the MS group and 3 of 163 (1.8%) in the MAC group (Fisher’s exact *p* = 0.741). Furthermore, all cRAS-positive patients in the MAC group met at least one operator-assessed criterion, and the three patients classified on pain criteria alone were all in the MS group. 

To evaluate whether the potent analgesic properties of the MAC regimen merely masked patient-reported symptoms, sensitivity analyses were conducted using outcome definitions with progressively lower reliance on pain (Table A2). When the definition was restricted solely to operator-assessed mechanical criteria, independent of patient report, the incidence of spasm remained significantly higher with standard moderate sedation (21.6% vs. 9.2%; *p* = 0.002). In multivariable modelling, the adjusted odds ratio for moderate sedation attenuated gradually from 3.69 to 2.95 across these increasingly stringent definitions, whilst remaining independently associated with the outcome (*p* = 0.001). The association lost statistical significance (OR: 1.66; 95% CI: 0.65 to 4.24; *p* = 0.289) only under the most restrictive endpoint, which strictly required the simultaneous presence of both operator-assessed criteria.

Any procedure-related pain (VAS ≥ 1) was reported by 132 of 231 patients (57.1%) with moderate sedation and 43 of 163 (26.4%) with MAC (Table 1). In univariate analysis, female sex, preprocedural calcium channel blocker use, prolonged procedure time, cRAS and moderate sedation were each associated with any procedure-related pain (all *p* < 0.05). In the subsequent multivariable model (Hosmer–Lemeshow *p* = 0.625, Nagelkerke R^2^ = 40.7%), the absence of cRAS emerged as the strongest protective factor against pain (OR: 0.030, 95% CI: 0.007–0.128; *p* < 0.001). Conversely, standard moderate sedation was associated with higher odds of any procedure-related pain (OR: 4.19, 95% CI: 2.47–7.10; *p* < 0.001). Female sex (OR: 1.85, 95% CI: 1.08–3.16; *p* = 0.024) and preprocedural calcium channel blocker use (OR: 1.98, 95% CI: 1.09–3.60; *p* = 0.024) remained significant independent risk factors (Table 5).

## 4. Discussion

In this prospective observational study, a propofol-integrated MAC regimen was associated with lower post-procedural pain scores and a lower incidence of clinically detected radial artery spasm compared with fentanyl-midazolam moderate sedation, with no difference in periprocedural complications. Two explanations are compatible with these data. First, the regimen may have reduced radial vasomotor reactivity; alternatively, it may have lowered the clinical detection of spasm, given that three of the five diagnostic criteria depend on patient-reported pain and the regimen directly alters pain perception, reporting, and recall. Both explanations describe an outcome that matters in practice: spasm is identified and managed in the cardiac catheterisation laboratory based on precisely these clinical grounds, and procedural pain is a patient-centred endpoint in its own right, independent of any vasomotor interpretation. The sensitivity analyses detailed in Section 4.1 indicate that the association is not confined to the pain-based components of the definition; however, they do not establish a true vasomotor mechanism.

Randomised trials have shown that sedation reduces radial spasm rates [5], an effect attributed to suppression of autonomic discharge in the catheterisation laboratory [7,9]. A vascular mechanism has been described for propofol in experimental work: enhancement of voltage-dependent inactivation of L-type calcium channels in vascular smooth muscle, and TRPA1-mediated endothelial nitric oxide release [10,11]. These findings provide a plausible basis for a peripheral vasomotor effect; however, no such effect was measured in the present study. Notably, the study did not include any assessment of radial artery diameter, flow, or vasomotor tone. These mechanisms are stated here as a hypothesis consistent with our observations rather than as an established explanation of them. While the isolated use of fentanyl and midazolam disrupts the autonomic reflex arc primarily through central anxiolysis and analgesia, it may not provide the direct and localised peripheral vasodilatory action required to break a severe myogenic spasm cycle triggered by local mechanical stimuli [8].

In standard clinical practice, localised radial hyperreactivity is traditionally managed with intra-arterial spasmolytics [8], or the application of modern non-invasive anti-spasm strategies, such as transdermal nitroglycerin patches [12]. In our study, all patients routinely received a universal intra-arterial cocktail comprising both nitroglycerin and diltiazem to suppress local vasoreactivity. Recent comparative data suggest that standalone nitrate administration may achieve equivalent spasmolytic efficacy without the transient diastolic hypotension associated with adjunctive calcium channel blockers [13]. Spasm nonetheless occurred despite uniform local prophylaxis, which is consistent with a contribution from systemic sympathetic tone. Whether the difference between groups reflects a peripheral vascular action, a difference in central sympathetic suppression, or a difference in the clinical detection of spasm cannot be determined from these data.

Rates of hypotension, bradycardia, respiratory depression and nausea or vomiting were similar between the moderate sedation and MAC groups. This finding is in contrast to the prevailing clinical concern that intravenous anaesthetic agents may induce uncontrolled haemodynamic fluctuations within the catheterisation laboratory environment [14,15]. It should not, however, be read as evidence of equivalent safety: the number of events was small, and the study was not designed to exclude a clinically relevant difference. What these data show is that propofol was added to a moderate sedation regimen without an observed excess of respiratory or haemodynamic events, under continuous anaesthesiologic monitoring and titration. 

Our study compares two multidrug sedation strategies rather than evaluating propofol on its own. Integrating propofol reduced the total fentanyl and midazolam requirements, which plausibly mitigates the risk of opioid-induced respiratory depression associated with higher opioid doses [16]. Simplified propofol-based regimens that reduce or omit adjunctive sedatives have been reported to maintain a favourable respiratory safety profile in other cardiac catheterisation laboratory settings [17].

In our study, female sex showed one of the strongest independent associations with the development of cRAS. Pathophysiologically, the increased propensity for spasm in females is primarily driven by their smaller radial artery lumen diameter compared to males, which predisposes them to an artery-sheath size mismatch [18]. The fact that the outer diameter of standard 6F sheaths exceeds the average anatomical thresholds of the radial artery in females may trigger endothelial nitric oxide synthase inhibition by generating continuous mechanical friction and tensile stress on the vessel wall [19,20]. This mechanical background, when coupled with the higher local catecholaminergic sensitivity observed in females, exacerbates the spasm cycle [21]. These considerations suggest that women may be a group in which sedation strategy and sheath size are worth evaluating prospectively, but the present data do not support a recommendation.

Women in this cohort were at higher risk of cRAS, and sex differences of this kind are well documented across the spectrum of coronary artery disease. Beyond stable elective cases, these differences in clinical profiles, treatment strategies, and outcomes are particularly evident in acute coronary syndromes. Specifically, although women presenting with NSTEMI have a higher burden of baseline comorbidities and undergo invasive testing less frequently, their overall mortality rates remain comparable to those of men [22]. Conversely, in the setting of STEMI, female gender is independently associated with significantly higher mortality following percutaneous coronary intervention [23]. These outcomes were not measured here and are cited only as context for why periprocedural risk in women matters. A sedation strategy that lowers this risk would therefore be of greatest potential value in this group.

Preoperative anxiety has been described as a trigger for radial artery spasm via sympathetic autonomic activation [24,25]. However, in the present cohort, pre-procedural VAS-Anxiety was not significantly associated with cRAS. This deviation from classical un-sedated cohorts can be primarily attributed to the systematic pharmacological approach used in this study. Given that all enrolled patients received early, protocol-driven administration of anxiolytic and sedative agents (midazolam-based regimens), the downstream translation of psychological stress into an acute localised sympathetic discharge was likely preemptively blunted [26]. Consequently, routine premedication appears to uncouple baseline anxiety from clinically detected spasm, reducing its value as an isolated risk factor in sedated cohorts.

Prolonged procedural duration was found to be independently associated with an increased risk of cRAS with every cumulative minute. Prolonged procedural durations result in increased contact between the catheter manipulations and the guide wire movements with the intravascular endothelial layer [27]. This, in turn, leads to the release of endothelin-1, a paracrine vasoconstrictor [28]. Although catheter exchanges were associated with cRAS in the univariate analysis, this parameter lost its independent predictive value in the multivariable model. This attenuation is likely attributable to collinearity with procedural time, as frequent catheter exchanges inherently prolong the total procedure duration, thereby indirectly exacerbating the risk of spasm [29]. The relationship between procedural time and cRAS is, however, bidirectional. While prolonged catheter manipulation triggers endothelial irritation and spasm, the occurrence of spasm itself mechanically impedes catheter movement, often necessitating additional maneuvers or catheter exchanges, and consequently prolonging the overall procedure. Due to the observational design of the study and the absence of precise time-stamping for the exact onset of spasm relative to the total procedural time, chronological causality cannot be definitively established. Consequently, procedural duration should be interpreted as a significant clinical correlate of a complex procedure complicated by spasm, rather than exclusively as a baseline predictor.

Beyond procedural comfort and local vascular dynamics, the choice of sedation regimen also affects the pharmacokinetics of oral antiplatelet agents, particularly during ad hoc percutaneous coronary intervention. Opioid derivatives delay gastrointestinal motility via the stimulation of mu-opioid receptors within the enteric nervous system, which consequently retards the absorption of oral P2Y12 inhibitors and leads to suboptimal platelet inhibition [30,31]. This pharmacokinetic limitation of opioid analgesia during percutaneous coronary interventions has prompted the investigation of alternative non-opioid sedation regimens, including nitrous oxide inhalation and tailored intravenous strategies, to maintain procedural comfort without compromising antiplatelet bioavailability [32]. Notably, achieving the Ramsay Sedation Scale score (2–3) in our MAC arm was possible with significantly lower doses of traditional agents due to the synergistic central nervous system depression provided by propofol. Consequently, this significant reduction in the required dose of fentanyl potentially offers a distinct clinical advantage by minimising the risk of opioid-induced gastric paralysis. The pharmacodynamic profile of propofol, which does not involve the opioid pathway, allows increased analgesic/sedative requirements to be met with propofol titration rather than escalating fentanyl doses [16]. This has the potential to contribute to the preservation of antiplatelet absorption safety.

The absence of cRAS was the strongest correlate of a pain-free procedure. This relationship is bidirectional. While overt spasm generates ischaemic and mechanical pain, acute nociceptive stimuli during catheter manipulation can induce a rapid sympathetic surge and localised catecholamine release, which subsequently triggers secondary reflex vasoconstriction [8,33]. Consequently, pain and spasm frequently function as part of a bidirectional ‘vicious cycle’. Given the observational design of our study, definitively establishing chronological causality between these two variables remains challenging. Because pain is an intrinsic component of the cRAS definition, incorporating cRAS as a predictor of post-procedural pain introduces definitional circularity. Consequently, this association should not be interpreted as an independent physiological pathway. Rather, cRAS was included in the model to test whether the sedation strategy influenced procedural comfort independently of spasm. After adjustment for cRAS, sex, procedural duration and calcium channel blocker use, moderate sedation remained associated with a higher odds of reporting any procedural pain. This is consistent with the propofol-integrated regimen having provided more effective overall sedation during sheath manipulation, rather than acting solely through a reduction in spasm. Propofol acts synergistically with opioids, and propofol-based regimens have been reported to achieve pain-free conditions in other catheter-based procedures with only low-dose opioid supplementation [17]; in the present cohort the target sedation level was likewise reached with lower fentanyl and midazolam doses. The comfort finding does not depend on resolving the question of mechanism: post-procedural pain was measured directly and was lower with MAC, whether or not the difference in cRAS reflects a change in vasomotor tone.

Female sex was independently associated with higher odds of pain, likely reflecting the aforementioned artery-sheath size mismatch that generates continuous frictional stress [18]. Preprocedural use of calcium channel blockers was also independently associated with an increased risk of pain. Rather than a direct pharmacological effect, this paradoxical association is likely due to ‘confounding by indication’. Patients requiring chronic CCB therapy often have a greater burden of advanced vascular disease, long-standing severe hypertension and significant underlying arterial stiffness [34]. These non-compliant vessels cannot easily accommodate the radial sheath. Therefore, the resulting mechanical stretch induces significant pain, which overrides any theoretical vasodilatory benefits of the medication [18].

### 4.1. Methodological Implications of the Clinical Spasm Definition

The clinical definition of radial artery spasm relies on patient-reported pain and operator-perceived resistance [8], a standard that performs adequately when sedation levels are held constant. A pharmacological agent that reduces pain perception lowers the likelihood of fulfilling symptom-based criteria; simultaneously, an agent that suppresses patient movement and sympathetic tone may diminish operator-perceived friction, independent of any true change in vessel calibre. A central concern raised by this diagnostic overlap is that MAC merely masked pain perception, thereby leading to the under-reporting of spasm. As detailed in the Results, progressively removing patient-reported pain from the diagnostic criteria reduced the adjusted odds ratio from 3.69 to 2.95, an attenuation of approximately 17% on the log-odds scale, and the association remained significant. The direction of this gradient is itself consistent with a contribution from differential detection, and we do not present it as evidence against that explanation. Had the observed difference arisen solely because MAC suppressed pain reporting, the endpoints excluding patient report would have been expected to converge; however, they did not. These endpoints are nonetheless independent of patient report rather than independent of sedation, and the operators were unblinded. Consequently, these sensitivity analyses narrow the range of alternative explanations without entirely excluding detection bias.

The magnitude of required bias can be quantified directly: assuming identical true prevalence of radial vasoconstriction and perfect diagnostic specificity, the clinical detection sensitivity in the MAC group would need to be 64% lower in relative terms than in the MS group to yield the observed rates of 7.4% versus 20.3%. A detection discrepancy of this magnitude should have left an identifiable trace among patients with operator-perceived resistance, as a substantial proportion of these individuals would have fallen below the two-criterion diagnostic threshold. However, the observed proportion falling below the threshold was only 1.8% in the MAC group and 2.6% in the MS group, and no patient receiving MAC was classified based on pain criteria alone. Consequently, bias of this magnitude is not evident in the segment of the cohort where it would be directly observable. Two limits apply to this rationale: first, only 15 patients in the MAC group exhibited an operator-assessed finding, which limits statistical power to distinguish between capture rates of 80% and 88%; second, true radial vasoconstriction that produced neither reported pain nor perceived resistance could not be detected by any metric available in this study.

Two distinct transitions occur across the outcome definitions and should not be read as one. Restricting the primary outcome to cases with at least one operator-assessed criterion removes the pain-based components without altering the clinical phenotype. Across this step, the adjusted odds ratio fell from 3.69 to 2.95 and remained significant. This indicates that the between-group difference does not depend entirely on patient-reported pain. A further restriction requiring both operator-assessed criteria removes no additional pain components. Rather, it shifts the identified phenotype from resistance at any single stage to resistance persisting across both catheter manipulation and sheath removal. The subsequent attenuation of the odds ratio to a non-significant 1.66 therefore reflects this transition toward a less frequent, more persistent clinical phenotype. An informative internal contrast is provided by the covariate estimates: the adjusted odds ratio for female sex remained stable across all four outcome definitions (2.98 to 3.61), whereas the estimate for sedation strategy declined monotonically. Within the same models and the same patients, an estimate that is insensitive to the outcome definition sits alongside one that is not, which indicates that the sedation estimate is in part definition-dependent, and we report it as such.

The difference between groups concerned spasm that was clinically apparent and transient rather than severe or sustained. The two operator-assessed criteria support this interpretation: resistance at sheath removal, which occurs once the procedure is complete, differed significantly between the groups, whereas spasmodic entrapment preventing catheter advancement did not. Similarly, crossover to transfemoral access, which represents the only endpoint requiring spasm severe enough to mandate procedural abort or site conversion, showed no significant difference between the cohorts. Operator unblinding also introduces potential bias into the subjective assessment of mechanical resistance. Although these analyses make a purely analgesic explanation less likely, separating symptom suppression from actual vasomotor relaxation requires objective metrics. Future studies must rely on blinded angiographic or ultrasonographic assessments of radial calibre to definitively resolve this distinction.

### 4.2. Limitations

Several methodological limitations should be acknowledged when interpreting the findings of this study. 

First, the clinical diagnosis of cRAS is open to differential misclassification: three of the five criteria depend on patient-reported pain, the remaining two on unblinded operator perception, and the intervention affects both. The post-procedural VAS likewise reflects recalled rather than real-time discomfort. Section 4.1 sets out the analyses bearing on this and their limits; they constrain the extent of such bias without excluding it.

Second, the single-centre, non-randomised observational design inherently introduces a substantial risk of confounding by indication. Treatment allocation was driven by operator discretion and the clinical judgment of the attending anaesthesia team rather than a systematic randomisation protocol. The decision to use MAC may have been influenced by unmeasured variables such as anticipated procedural difficulty, previous procedural experiences, patient cooperation, or the immediate availability of the anaesthesia team. While we adjusted for measured baseline characteristics via multivariable regression, this statistical approach cannot account for these unmeasured confounding factors. Therefore, causality between the sedation strategy and the reduction in cRAS cannot be definitively established based on these observational data alone. 

Third, the diagnosis of spasm was based on qualitative clinical criteria rather than intravascular high-resolution imaging modalities. No imaging or physiological measure of radial calibre, flow or vasomotor tone was obtained at any point, so the presence of true vasospasm was never verified in any patient, including those classified as positive. Consequently, coexisting micro-intimal dissection flaps may have been overlooked or underestimated in some cases. 

Fourth, while pharmacological agents were titrated to clinical response targeting a Ramsay Sedation Scale score of 2 to 3, serial sedation scores were not systematically logged during the procedure. As a result, exact fluctuations in sedation depth across different procedural time points cannot be retrospectively compared between the groups. To mitigate this potential confounding, patients requiring general anaesthesia or deep sedation necessitating airway instrumentation were strictly excluded from the study, as outlined in our predefined criteria. Furthermore, the necessity for patients to actively report intraprocedural pain and complete the post-procedural VAS indicates that sustained deep sedation was clinically avoided. However, in the absence of serial sedation scoring, we cannot rule out that the MAC strategy provided a more stable depth of sedation compared to the MS regimen, which may have independently contributed to the observed reduction in cRAS. 

Fifth, the baseline radial artery diameter and the precise sheath-to-artery ratio were not systematically measured via pre-procedural ultrasound. As this was a real-world observational registry, pre-procedural ultrasound imaging was not part of the standard institutional protocol. Consequently, our mechanistic explanation regarding the artery-sheath mismatch in female patients is an inference drawn from the literature, rather than a directly measured correlation in our cohort. Similarly, data regarding patients’ previous TRA history were not systematically queried, representing an additional unmeasured variable. 

Sixth, our study focused exclusively on acute periprocedural outcomes and lacked follow-up data regarding radial artery occlusion. Recent comprehensive meta-analyses have demonstrated the efficacy of vasodilator prophylaxis in preventing both acute spasm and subsequent RAO [35]. Given this established pathophysiological link, the absence of long-term follow-up to assess whether the lower incidence of cRAS observed with MAC also translates to lower RAO rates is a notable clinical limitation.

Finally, this study was not statistically powered as a formal non-inferiority trial to definitively evaluate comparative safety. Although no significant difference was observed in secondary adverse events, the relatively small number of complications limits our ability to conclusively assert equivalent safety between the two strategies. 

## 5. Conclusions

In this observational study, a propofol-integrated monitored anaesthesia care regimen was associated with reduced procedural pain and a lower incidence of clinically detected radial artery spasm compared with standard moderate sedation, with no observed excess of adverse events. These hypothesis-generating findings reflect clinically apparent, transient spasm rather than the prevention of true radial vasospasm, and the study was not powered for comparative safety endpoints. Within these limits, integrating propofol into a moderate sedation regimen was associated with greater procedural comfort and a reduced incidence of the spasm that operators clinically encounter and manage. Women had the highest rate of cRAS in this cohort. These findings identify the strategy and the population that an adequately powered randomised trial with objective, blinded endpoints should now test.

## Figures and Tables

**Table 1:** Distribution of Demographic, Clinical, and Procedural Characteristics of Patients According to Sedation Type.

Variable	All Patients (*n* = 394)	Moderate Sedation (*n* = 231)	MAC (*n* = 163)	*p*
	Median (Q1–Q3) or *n* (%)	Median (Q1–Q3) or *n* (%)	Median (Q1–Q3) or *n* (%)	
Demographic Characteristics				
Age (years)	63.0 (56.0–69.0)	64.0 (57.0–70.0)	62.0 (55.0–68.0)	0.057
BMI (kg/m^2^)	27.9 (25.1–31.4)	27.6 (24.9–31.6)	28.3 (25.7–31.2)	0.559
Female Sex, *n* (%)	136 (34.5)	78 (33.8)	58 (35.6)	0.790
Smoking status				0.516
Active	150 (38.1)	87 (37.7)	63 (38.7)	
Former	113 (28.7)	71 (30.7)	42 (25.8)	
Never smoked	131 (33.2)	73 (31.6)	58 (35.6)	
Clinical and Vital Signs				
ASA Score				0.897
1	21 (5.3)	12 (5.2)	9 (5.5)	
2	366 (92.9)	214 (92.6)	152 (93.3)	
3	7 (1.8)	5 (2.2)	2 (1.2)	
Heart rate (bpm)	80.0 (70.0–88.0)	80.0 (70.0–88.0)	80.0 (70.0–88.0)	0.743
Systolic BP (mmHg)	130.0 (120.0–140.0)	130.0 (120.0–140.0)	130.0 (120.0–140.0)	0.646
Diastolic BP (mmHg)	70.0 (70.0–80.0)	70.0 (70.0–80.0)	73.0 (68.0–80.0)	0.691
O_2_ Saturation (%)	97.0 (96.0–98.0)	96.0 (95.0–98.0)	97.0 (96.0–98.0)	0.001
Pre-procedural VAS-Anxiety	4.0 (2.0–6.0)	4.0 (2.0–6.0)	4.0 (3.0–6.0)	0.211
Drug Dosages				
Midazolam dose (mg)	1.5 (1.0–1.8)	1.5 (1.2–1.8)	1.0 (1.0–1.1)	<0.001
Fentanyl dose (mcg)	35.0 (25.0–40.0)	40.0 (35.0–45.0)	20.0 (20.0–25.0)	<0.001
Propofol dose (mg)	-	0.0 (0.0–0.0)	40.0 (30.0–50.0)	-
I.A. nitrate dose (mcg)	300.0 (300.0–700.0)	300.0 (300.0–700.0)	300.0 (300.0–600.0)	0.527
Heparin dose (U)	5000 (5000–7500)	5000 (5000–7500)	5000 (5000–7500)	0.744
Procedural Characteristics				
Number of punctures	1.0	1.0	1.0	0.823
Number of catheters	2.0 (2.0–3.0)	2.0 (2.0–3.0)	2.0 (2.0–3.0)	0.272
Number of catheter exchanges	1.0 (1.0–2.0)	1.0 (1.0–2.0)	1.0 (1.0–2.0)	0.253
Procedure time (min)	15.0 (10.0–25.0)	17.0 (10.0–25.0)	15.0 (10.0–25.0)	0.844
Right radial access, *n* (%)	379 (96.2)	224 (97.0)	155 (95.1)	0.354
Crossover to femoral, *n* (%)	10 (2.5)	6 (2.6)	4 (2.5)	1.000
Post-procedural VAS-Pain	0.0 (0.0–2.0)	1.0 (0.0–2.0)	0.0 (0.0–1.0)	<0.001
Any procedure-related pain (VAS ≥ 1), *n* (%)	175 (44.4)	132 (57.1)	43 (26.4)	<0.001
Comorbidities and Pre-procedural Drug Use				
Hypertension, *n* (%)	233 (59.1)	143 (61.9)	90 (55.2)	0.220
Diabetes mellitus, *n* (%)	141 (35.8)	84 (36.4)	57 (35.0)	0.859
Dyslipidemia, *n* (%)	98 (24.9)	62 (26.8)	36 (22.1)	0.339
Peripheral arterial disease, *n* (%)	8 (2.0)	5 (2.2)	3 (1.8)	1.000
Coronary artery disease, *n* (%)	156 (39.6)	89 (38.5)	67 (41.1)	0.682
Beta-blocker use, *n* (%)	45 (11.4)	28 (12.1)	17 (10.4)	0.719
Calcium channel blocker, *n* (%)	303 (76.9)	180 (77.9)	123 (75.5)	0.653
Nitrate use, *n* (%)	291 (73.9)	176 (76.2)	115 (70.6)	0.255
Type of procedure				0.503
Diagnostic CAG, *n* (%)	272 (69.0)	163 (70.6)	109 (66.9)	
Ad hoc PCI, *n* (%)	122 (31.0)	68 (29.4)	54 (33.1)	

Abbreviations: ASA, American Society of Anesthesiologists; BMI, body mass index; BP, blood pressure; bpm, beats per minute; CAG, coronary angiography; I.A., intra-arterial; MAC, monitored anaesthesia care; PCI, percutaneous coronary intervention; Q1, first quartile; Q3, third quartile; VAS, Visual Analogue Scale. Note: Propofol was administered in the MAC group only; no between-group comparison is reported for this variable.

**Table 2:** Comparison of Variables According to the Development of cRAS.

Variable	No cRAS (*n* = 335)	cRAS Present (*n* = 59)	*p*
	Median (Q1–Q3) or *n* (%)	Median (Q1–Q3) or *n* (%)	
Demographic Characteristics			
Age (years)	63.0 (56.0–69.5)	62.0 (49.5–68.0)	0.204
BMI (kg/m^2^)	27.8 (24.9–31.2)	28.7 (26.4–33.0)	0.237
Female Sex, *n* (%)	103 (30.7)	33 (55.9)	<0.001
Smoking Status			0.465
Active	126 (37.6)	24 (40.7)	
Former	100 (29.9)	13 (22.0)	
Never smoked	109 (32.5)	22 (37.3)	
Clinical and Vital Signs			
ASA Score			0.092
1	19 (5.7)	2 (3.4)	
2	312 (93.1)	54 (91.5)	
3	4 (1.2)	3 (5.1)	
Heart rate (bpm)	80.0 (70.0–88.0)	80.0 (70.0–88.0)	0.919
Systolic BP (mmHg)	130.0 (120.0–140.0)	130.0 (120.0–140.0)	0.140
Diastolic BP (mmHg)	72.0 (70.0–80.0)	70.0 (70.0–80.0)	0.554
O_2_ Saturation (%)	97.0 (96.0–98.0)	97.0 (96.0–98.0)	0.915
Pre-procedural VAS-Anxiety score	4.0 (2.0–6.0)	5.0 (3.0–6.0)	0.057
Post-procedural VAS-Pain score	0.0 (0.0–1.0)	3.0 (2.0–5.0)	<0.001
Comorbidities and Drug Use			
Hypertension, *n* (%)	198 (59.1)	35 (59.3)	1.000
Diabetes mellitus, *n* (%)	120 (35.8)	21 (35.6)	1.000
Dyslipidemia, *n* (%)	78 (23.3)	20 (33.9)	0.115
Peripheral arterial disease, *n* (%)	7 (2.1)	1 (1.7)	1.000
Coronary artery disease, *n* (%)	131 (39.1)	25 (42.4)	0.742
Beta-blocker use, *n* (%)	36 (10.7)	9 (15.3)	0.434
Calcium channel blocker, *n* (%)	260 (77.6)	43 (72.9)	0.530
Nitrate use, *n* (%)	248 (74.0)	43 (72.9)	0.980
Type of Procedure			0.496
Diagnostic CAG, *n* (%)	234 (69.9)	38 (64.4)	
Ad hoc PCI, *n* (%)	101 (30.1)	21 (35.6)	
Number of Punctures			0.200
1	283 (84.5)	45 (76.3)	
2	38 (11.3)	11 (18.6)	
3 or more	14 (4.2)	3 (5.1)	
Number of Catheters	2.0 (2.0–3.0)	3.0 (2.0–3.0)	0.068
1	47 (14.0)	5 (8.5)	
2	147 (43.9)	24 (40.7)	
3	126 (37.6)	24 (40.7)	
4 or more	15 (4.5)	6 (10.2)	
Number of Catheter Exchanges	1.0 (1.0–2.0)	2.0 (1.0–2.0)	0.042
0	45 (13.4)	4 (6.8)	
1	143 (42.7)	25 (42.4)	
2	127 (37.9)	21 (35.6)	
3 or more	20 (6.0)	9 (15.3)	
Procedure Time (min)	15.0 (10.0–25.0)	25.0 (15.0–35.0)	<0.001
Sedation Type			0.001
Moderate Sedation, *n* (%)	184 (54.9)	47 (79.7)	
MAC, *n* (%)	151 (45.1)	12 (20.3)	

Abbreviations: ASA, American Society of Anesthesiologists; BMI, body mass index; BP, blood pressure; bpm, beats per minute; CAG, coronary angiography; MAC, monitored anaesthesia care; PCI, percutaneous coronary intervention; Q1, first quartile; Q3, third quartile; cRAS, clinically detected radial artery spasm; VAS, visual analogue scale.

**Table 3:** Comparison of Specific Secondary Complications According to Sedation Type.

	MS (*n* = 231) *n* (%)	MAC (*n* = 163) *n* (%)	*p* Value
Composite Secondary Outcomes *	31 (13.4)	12 (7.3)	0.083
Hypotension	0 (0.0)	2 (1.2)	0.171
Bradycardia	4 (1.7)	2 (1.2)	1.000
Respiratory Depression	27 (11.7)	11 (6.7)	0.102
Nausea/Vomiting	1 (0.4)	1 (0.6)	1.000

Abbreviations: MAC, monitored anaesthesia care; MS, Moderate Sedation. Note: *p*-values for 2 × 2 comparisons were calculated using the chi-square test with Yates’ continuity correction, and Fisher’s exact test when any expected cell count was below five; comparisons across more than two categories used Pearson’s chi-square test. * Some patients may have experienced more than one complication simultaneously.

**Table 4:** Multivariable Logistic Regression Analysis of Factors Associated with Clinically Detected Radial Artery Spasm.

Variable	Odds Ratio (OR)	95% Confidence Interval (CI)	*p* Value
Female Sex	3.601	1.912–6.780	<0.001
Procedure time	1.043	1.020–1.067	<0.001
Sedation type (MS vs. MAC)	3.695	1.800–7.586	<0.001
Number of catheter exchanges			0.309
Exchange count (1)	1.695	0.490–5.865	0.404
Exchange count (2)	1.478	0.443–4.929	0.525
Exchange count (3 or more)	2.576	0.615–10.783	0.195

Abbreviations: CI, confidence interval; MAC, monitored anaesthesia care; MS, moderate sedation; OR, odds ratio.

**Table 5:** Multivariable Logistic Regression Analysis of Factors Associated with Post-Procedural Pain.

Variable	Odds Ratio (OR)	95% Confidence Interval	*p* Value
Absence of cRAS	0.030	0.007–0.128	<0.001
Sedation Type (Moderate Sedation vs. MAC)	4.190	2.471–7.104	<0.001
Female Sex	1.852	1.085–3.162	0.024
Pre-procedural CCB Use	1.986	1.094–3.604	0.024
Age	0.975	0.950–1.001	0.061
Dyslipidemia	0.581	0.327–1.031	0.063
Procedure Time (per 1 min increase)	1.021	0.999–1.044	0.065

Abbreviations: CCB, calcium channel blocker; MAC, monitored anaesthesia care.

## Data Availability

The data presented in this study are available on request from the corresponding author. The data are not publicly available due to privacy and ethical restrictions regarding the protection of personal health information of the participants.

## Abbreviations

The following abbreviations are used in this manuscript:

ASA	American Society of Anesthesiologists
BMI	Body mass index
BP	Blood pressure
bpm	Beats per minute
CAG	Coronary angiography
CCB	Calcium channel blocker
CI	Confidence interval
ECG	Electrocardiography
I.A.	Intra-arterial
IQR	Interquartile range
IV	Intravenous
MAC	Monitored anaesthesia care
MS	Moderate sedation
OR	Odds ratio
PCI	Percutaneous coronary intervention
Q1	First quartile
Q3	Third quartile
RAS	Radial artery spasm
cRAS	Clinically detected radial artery spasm
RAO	Radial Artery Occlusion
TRA	Transradial coronary angiography
TRPA1	Transient receptor potential ankyrin 1
VAS	Visual Analogue Scale

## Appendix A

**Table A1:** Frequency of Each Diagnostic Criterion For Clinically Detected Radial Artery Spasm, by Sedation Strategy.

Criterion	Type	MS (*n* = 231)	MAC (*n* = 163)	OR (95% CI)	*p*
1. Persistent forearm pain throughout the procedure	Patient-reported	5 (2.2)	1 (0.6)	3.58 (0.41–30.97)	0.408
2. Forearm pain only during catheter or guidewire manipulation	Patient-reported	19 (8.2)	3 (1.8)	4.78 (1.39–16.43)	0.013
3. Pain during sheath insertion or removal	Patient-reported	63 (27.3)	17 (10.4)	3.22 (1.80–5.75)	<0.001
4. Spasmodic entrapment of catheter or guidewire	Operator-assessed	26 (11.3)	10 (6.1)	1.94 (0.91–4.14)	0.119
5. Increased resistance during sheath removal	Operator-assessed	40 (17.3)	12 (7.4)	2.64 (1.34–5.20)	0.007

Note: Values are *n* (%). Odds ratios are for MS versus MAC. *p*-values were calculated using the chi-square test with Yates’ continuity correction, except for criterion 1, where the expected cell count was below five and Fisher’s exact test is reported. Criteria are not mutually exclusive; a patient meeting at least two was classified as cRAS-positive.

**Table A2:** Association Between Sedation Strategy and Clinically Detected Radial Artery Spasm Under Outcome Definitions with Decreasing Dependence on Patient-Reported Pain.

Outcome Definition	Pain-Based Component	Phenotype	MS (*n* = 231)	MAC (*n* = 163)	*p*	Adjusted OR, Sedation	Adjusted OR, Female Sex	Nagelkerke R^2^
Clinically detected RAS, ≥2 of 5 criteria (primary)	Three of five criteria	Any	47 (20.3)	12 (7.4)	0.001	3.69 (1.80–7.58)	3.60 (1.91–6.78)	0.216
cRAS with ≥1 operator-assessed criterion	Partial	Any	44 (19.0)	12 (7.4)	0.002	3.31 (1.62–6.76)	3.61 (1.93–6.76)	0.204
Either operator-assessed criterion	None	Any	50 (21.6)	15 (9.2)	0.002	2.95 (1.55–5.59)	2.98 (1.68–5.29)	0.162
Both operator-assessed criteria	None	Resistance at both stages	16 (6.9)	7 (4.3)	0.379	1.66 (0.65–4.24)	3.31 (1.34–8.13)	0.122

Note: Odds ratios are reported for MS versus MAC and for female versus male sex, mutually adjusted and further controlled for procedural duration. *p*-values for 2 × 2 comparisons were calculated using the chi-square test with Yates’ continuity correction, and Fisher’s exact test when any expected cell count was below five; comparisons across more than two categories used Pearson’s chi-square test. The final two endpoints are indicators of operator-perceived mechanical resistance that are independent of patient report, and should not be interpreted as alternative definitions of cRAS. Because the two operator-assessed criteria arise at different procedural stages, requiring both identifies mechanical resistance present both during catheter manipulation and at sheath removal rather than two concurrent signs.
